# Oral Biopsy: A Dental Gawk

**DOI:** 10.4103/2006-8808.73627

**Published:** 2010

**Authors:** Rajiv Saini, Santosh Saini, Sugandha Sharma

**Affiliations:** *Department of Periodontology & Oral Implantology, Rural Dental College, Loni, Maharashtra, India*; 1 *Department of Microbiology, Rural Medical College, Loni, Maharashtra, India*; 2 *Department of Prosthodontics, Rural Dental College, Loni, Maharashtra, India. E-mail: drperiodontist@yahoo.co.in*

Sir,

Dermatologists are often confronted with neoplasms and diseases of the oral cavity. Although many may be reluctant to perform oral surgical procedures, a biopsy is often needed to establish a definitive diagnosis, and biopsy of the oral cavity is a safe and useful technique that can be easily employed by dermatologists. Biopsies should be kept superficial, and neurovascular structures must be avoided to prevent complications. The primary step in oral cancer detection and diagnosis is patient history and thorough soft-tissue examination. If a suspicious lesion is discovered, it is biopsied and a histological examination of the sample is performed. Currently, a biopsy with histopathology is considered the gold standard for diagnosis of squamous cell carcinoma. The word biopsy originates from the Greek terms bios (life) and opsis (vision): vision of life. A biopsy consists of the obtainment of tissue from a living organism with the purpose of examining it under the microscope, in order to establish a diagnosis based on the sample.[1] There are oral lesions whose diagnosis can be made relying on data gathered during the history and/or physical examination, but there are others where histopathological studies are needed to confirm the presumed clinical diagnosis. The aim of biopsy is to define a lesion on the basis of its histopathological aspect, to establish a prognosis in malignant or premalignant lesions, facilitate the prescription of specific treatment, contribute to the assessment of the efficacy of the treatment, and act as a document with medical-legal value. Thorough inspection of the oral cavity should be a part of any complete head and neck examination. Close visual inspection should be performed with adequate lighting, and each of the anatomic regions in the oral cavity should be palpated. Approximately 10% of patients who are examined have some abnormality of the oral mucosa. Some lesions can be diagnosed on the basis of the history and clinical findings alone. For other lesions, additional information may be required to properly guide any indicated therapy. Often, biopsy with ample tissue for microscopic analysis is the definitive procedure. Numerous methods can be used to collect tissue samples from the oral mucosa for histopathologic examination. Performing biopsy with a scalpel is the standard and generally produces the most satisfactory specimen. Other techniques include the use of a needle, biopsy punch, biopsy forceps, laser, or electrocautery device. Needles may be appropriate in sampling cells from mass lesions, but they are of no benefit in the evaluation of surface lesions. Electrocautery produces thermal damage and artifact, which make evaluation of the specimen difficult; therefore, electrosurgery should be avoided during oral mucosal biopsy. Electrosurgery may be of benefit for wide local excisions of known intraoral malignancies after a scalpel is used to atraumatically obtain marginal specimens for frozen sections. Oral biopsy is considered essential for the definitive diagnosis of diseases of the oral mucosa, and for the subsequent planning of appropriate treatment. Although the obtainment of biopsies is widely used in all medical fields, the practice is not so widespread in dental practice—fundamentally because of a lack of awareness of the procedure among dental professionals. In this context, it must be taken into account that the early diagnosis of invasive oral malignancy may be critical for improving the patient prognosis.[1,2] When considering biopsy, a little forward planning and thought can greatly improve the diagnostic value obtained. Careful handling of the tissue and prompt appropriate fixation will enable a confident histological diagnosis to be reached. Inadequate care at any stage could result in a nondiagnostic biopsy and may necessitate the patient having a repeat procedure with its ensuing physical and psychological morbidity.[2] Lastly, the screening tool of greatest benefit is cautious oral examination and sound clinical judgment. The clinician’s knowledge and training may eliminate the need for biopsy in cases where lesions are clinically definable. For entities of uncertain significance or etiology, a biopsy provides the simplest and most speedy means of obtaining the perfect diagnosis. In the concern of the patient‘s welfare, correct diagnosis is of extreme importance.
